# Cross-mating between the alien bumblebee *Bombus terrestris* and two native Japanese bumblebees, *B. hypocrita sapporensis* and *B. cryptarum florilegus*, in the Nemuro Peninsula, Japan

**DOI:** 10.1038/s41598-023-38631-7

**Published:** 2023-07-17

**Authors:** Ryohei Kubo, Yuine Asanuma, Erina Fujimoto, Hisashi Okuyama, Masato Ono, Jun-ichi Takahashi

**Affiliations:** 1 Honeybee Science Research Center, Tamagawa University, 6-1-1, Tamagawagakuen, Machida, Tokyo, Japan; 2 Faculty of Life Sciences, Kyoto Sangyo University, Kamigamo, Motoyama, Kita-Ku, Kyoto, Japan

**Keywords:** Ecology, Zoology

## Abstract

The rapid naturalization of *Bombus terrestris* across the Nemuro Peninsula has led to a decline in two closely related native Japanese species, namely *Bombus hypocrita sapporensis* and *Bombus cryptarum florilegus*, both belonging to the common subgenus *Bombus*. Although it is widely believed that cross-mating of native and non-native species is influenced by the common male sex pheromone in this region, no study has been conducted to substantiate this claim. Thus, we investigated the cross-activities of male sex pheromones between native and non-native bumblebees, as well as the frequencies of cross-mating, using chemical and DNA assays. Our gas chromatography–electroantennographic detector analyses and behavioral tests revealed the presence of sex pheromonal cross-activities between *B. terrestris* and the two Japanese bumblebees species. Furthermore, DNA analyses revealed the occurrence of cross-mating between native and non-native species in the Nemuro Peninsula. Overall, these results indicate the immediate need for conservation measures to safeguard Japanese bumblebee populations in the Nemuro Peninsula.

## Introduction

Since its designation as an alien species in Japan in 2006, the imported bumblebee *Bombus terrestris* L. has posed significant challenges. Although *B. terrestris* serves as an important greenhouse pollinator, escaped queens and males have established feral populations in Hokkaido, Japan. This naturalization process has led to competition between *B. terrestris* and native Japanese bumblebee species, the disruption of symbiotic relationships between native plants and bumblebees, and the introduction of new pests and pathogens^1–8^.

Under laboratory conditions, hybridization between *B. terrestris* and closely related Japanese bumblebee species, such as *B. hypocrita hypocrita*, *B. h. sapporensis*, and *B. ignitus*, occurs readily^9^. However, no viable hybrids are produced when native queens mate with *B. terrestris* males, as the laid eggs cease embryonic development^10,11^. Nevertheless, cross-mating between these species has detrimental effects on the reproduction of native bumblebees, as *Bombus* queens typically mate only once or twice^12^. Furthermore, previous DNA analysis revealed the presence of *B. terrestris* spermatozoa in the spermathecae of wild queens from *B. h. hypocrita*, *B. h. sapporensis*, and *B. ignitus*, indicating that cross-mating between *B. terrestris* males mate and native queens occurs in the field^11^. Our previous study also suggested that cross-mating is facilitated by the similarities in male sex pheromone production in the labial gland (LG)^13^.

The Nemuro Peninsula, located in eastern Hokkaido, represents a valuable habitat for native bumblebees, with 10 out of 15 Japanese species, including the rare bumblebee species, *B. cryptarum florilegus*, thriving in this region^14^. Indeed, *B. c. florilegus* exhibits a restricted distribution, occurring only in the Nemuro and Notsuke Peninsulas in Japan, and is listed as a near-threatened species in the Japan Red List^14^. Given that *B. terrestris* has naturalized in this region, the populations of *B. h. sapporensis* and *B. c. florilegus* have been steadily declining^15^. Although it is widely believed that cross-mating between native and non-native species is influenced by the common male sex pheromone in the region, there is no empirical evidence to support this hypothesis. Therefore, using chemical and DNA assays, we investigated the frequencies of cross-mating and the cross-activities of male sex pheromones between native and non-native bumblebees.

## Results

**Figure 1:**
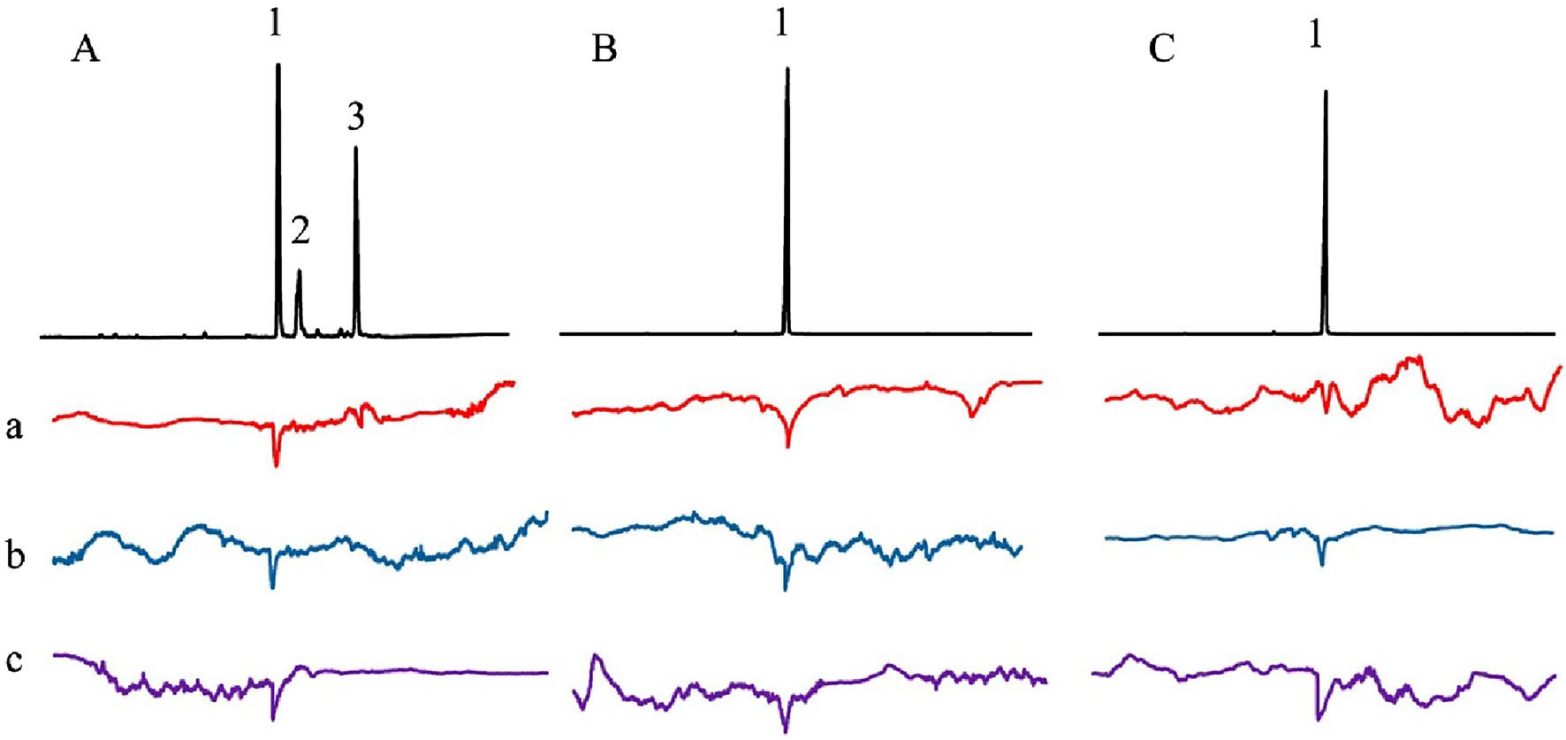
Simultaneous gas chromatography–flame ionization detector and electroantennographic detector recordings from (a) *Bombus terrestris*, (b) *B. hypocrita sapporensis*, and (c) *B. cryptarum florilegus* virgin queens using volatiles collected from the male labial gland (LG) of (**A**) *B. terrestris*, (**B**) *B. h. sapporensis,* and (**C**) *B. c. florilegus*. Identified compounds: (1) ethyl dodecanoate; (2) 2,3-dihydrofarnesal; and (3) 2,3-dihydrofarnesol.

Previous gas chromatography–electroantennographic detector (GC-EAD) analyses confirmed the presence of ethyl dodecanoate, 2,3-dihydrofarnesal, and 2,3-dihydrofarnesol emitted by the male LG in *B. terrestris*^16^. Additionally, ethyl dodecanoate has been detected in the male LG of *B. h. sapporensis*^13^. Our GC-EAD analyses confirmed that the male LG of *B. c. florilegus* emits ethyl dodecanoate. In addition, ethyl dodecanoate and 2,3-dihydrofarnesol elicited clear electrophysiological antennal responses in *B. terrestris* virgin queens (Fig. 1Aa). However, only ethyl dodecanoate evoked clear electrophysiological antennal responses in *B. h. sapporensis* and *B. c. florilegus* virgin queens (Fig. 1Ab,Ac,Ba,Bb,Bc,Ca,Cb,Cc).

**Figure 2:**
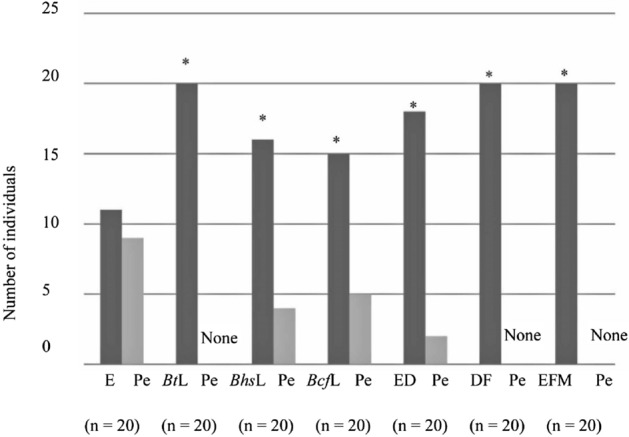
Attractiveness of labial gland (LG) extract scents from *Bombus terrestris* (*Bt*L), *B. h. sapporensis* (*Bhs*L), and *B. c. florilegus* (*Bcf*L), as well as ethyl dodecanoate (ED), 2,3-dihydrofarnesol (DF), and a mixture of ethyl dodecanoate and 2,3-dihydrofarnesol (EFM), against the solvent pentane (Pe) and an empty control (E) in *Bombus terrestris* virgin queens. The *y*-axis indicates the number of individuals choosing each scent within 5 min. Asterisks indicate statistical significance (*P* < 0.05) determined through binomial tests.

**Figure 3:**
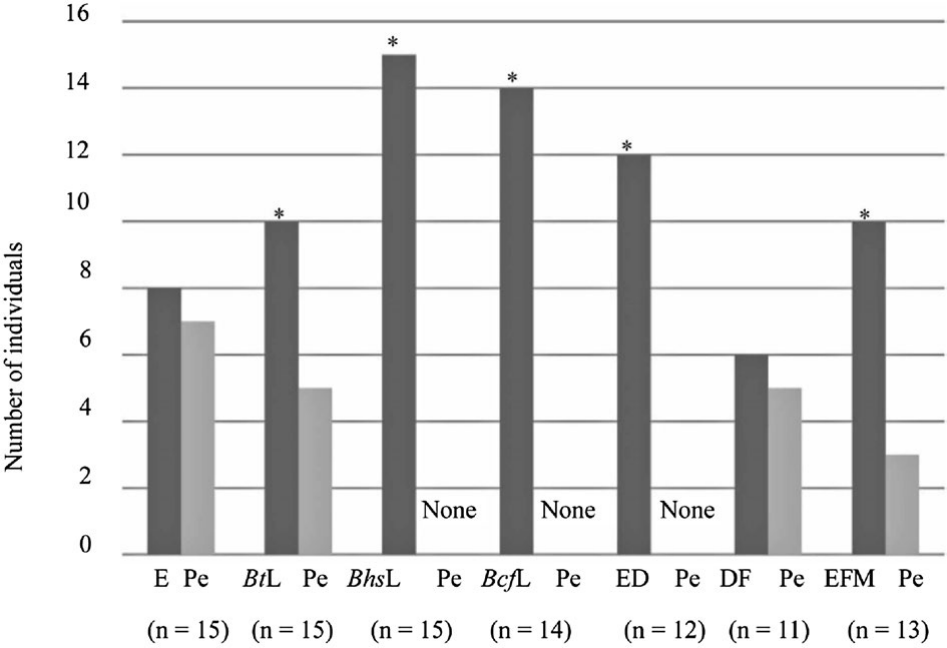
Attractiveness of scents from labial gland (LG) extracts from *Bombus terrestris* (*Bt*L), *B. h. sapporensis* (*Bhs*L), and *B. c. florilegus* (*Bcf*L), as well as ethyl dodecanoate (ED), 2,3-dihydrofarnesol (DF), and a mixture of ethyl dodecanoate and 2,3-dihydrofarnesol (EFM), against the solvent pentane (Pe) and an empty control (E) in *B. h. sapporensis* virgin queens. The *y*-axis indicates the number of individuals choosing either scent within 5 min. Asterisks indicate statistical significance (*P* < 0.05) determined through binomial tests.

**Figure 4:**
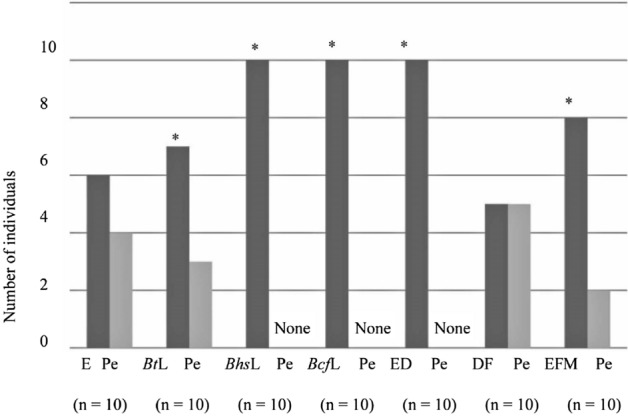
Attractiveness of scents from labial gland (LG) extracts from *Bombus terrestris* (*Bt*L), *B. h. sapporensis* (*Bhs*L), and *B. c. florilegus* (*Bcf*L), as well as ethyl dodecanoate (ED), 2,3-dihydrofarnesol (DF), and a mixture of ethyl dodecanoate and 2,3-dihydrofarnesol (EFM), against the solvent pentane (Pe) and an empty control (E) in *B. c. florilegus* virgin queens. The *y*-axis indicates the number of individuals choosing either scent within 5 min. Asterisks indicate statistical significance (*P* < 0.05) determined through binomial tests.

Binomial test analysis of behavioral tests conducted using a Y-tube olfactometer revealed that *B. terrestris* queens (n = 20) showed a significant (*P* < 0.05) preference for *Bt*L (*P* = 0.000067), *Bhs*L (*P* = 0.023103), *Bcf*L (*P* = 0.044357), ED (*P* = 0.00109), DF (*P* = 0.000067), and EFM (*P* = 0.000067) over pentane (Fig. 2). Similarly, based on binomial test analysis, *B. h. sapporensis* queens exhibited a significant (*P* < 0.05) preference for *Bt*L (*P* = 0.041656, n = 15), *Bhs*L (*P* = 0.000214, n = 15), *Bcf*L (*P* = 0.000366, n = 14), ED (*P* = 0.001221, n = 12), and EFM (*P* = 0.034912, n = 13) over pentane (Fig. 3). Likewise, binomial test analysis revealed that *B. c. florilegus* queens (n = 10) showed a significant (*P* < 0.05) preference for *Bt*L (*P* = 0.017578), *Bhs*L (*P* = 0.006836), *Bcf*L (*P* = 0.006836), ED (*P* = 0.006836), and EFM (*P* = 0.043945) over pentane (Fig. 4). However, neither *B. h. sapporensis* (n = 11) nor *B. c. florilegus* queens (n = 10) exhibited attraction toward or repulsion from DF compared with pentane (binomial test, *P* = 0.225586 and *P* = 0.246094, respectively) (Figs. 3 and 4). Furthermore, *B. terrestris* (n = 20), *B. h. sapporensis* (n = 15), and *B. c. florilegus* (n = 10) queens exhibited no attraction toward or repulsion from pentane compared with an empty control (binomial test, *P* = 0.160179, *P* = 0.196381, and *P* = 0.205078, respectively) (Figs. 2, 3, and 4).

Mitochondrial DNA cytochrome c oxidase subunit I (*COXI*) gene sequences extracted from the sperm-derived DNA were found to have a 100% match with the sequences registered for each of the three species in the DNA Data Bank of Japan (LC695022, LC695025, and LC695021). We successfully decoded the *ITS2* region of the three bumblebee species, which exhibited species-specific mutations. The *ITS2* sequences of these species were registered in the DNA Data Bank of Japan (LC769002–LC769004). Notably, the *ITS2* sequences obtained from sperm-derived DNA in the spermathecae of several queens showed a different species identification. The identification of cross-mating queens based on the *ITS2* region was in agreement with the results of mitochondrial DNA analysis.

The results obtained from species-specific polymerase chain reaction (PCR) and sequence analysis were consistent, revealing that 9.9% of *B. c. florilegus* queens in the Nemuro Peninsula were inseminated by invading *B. terrestris* males. Additionally, 4.4% of *B. h. sapporensis* queens stored sperm from *B. terrestris* males in their spermathecae. Conversely, we observed no interbreeding between *B. terrestris* queens and the males of native species. Through dissection, we confirmed the absence of male sperm in the spermathecae of some queens of the three species. The frequency of unmated queens was found to be was 13.5%, 2.8%, and 2.8% for *B. c. florilegus*, *B. h. sapporensis,* and *B. terrestris* respectively (Table 1).

**Table 1:** Frequencies of intraspecific and interspecific mating in queens of three *Bombus* species.

Queen species	*n*	Frequency of interspecific mating in queen	References
*Bombus cryptarum florilegus*	141	0.099	Present study
*Bombus terrestris*	250	0.000	Present study
*Bombus hypocrita sapporensis*	250	0.044	Present study

**Figure 5:**
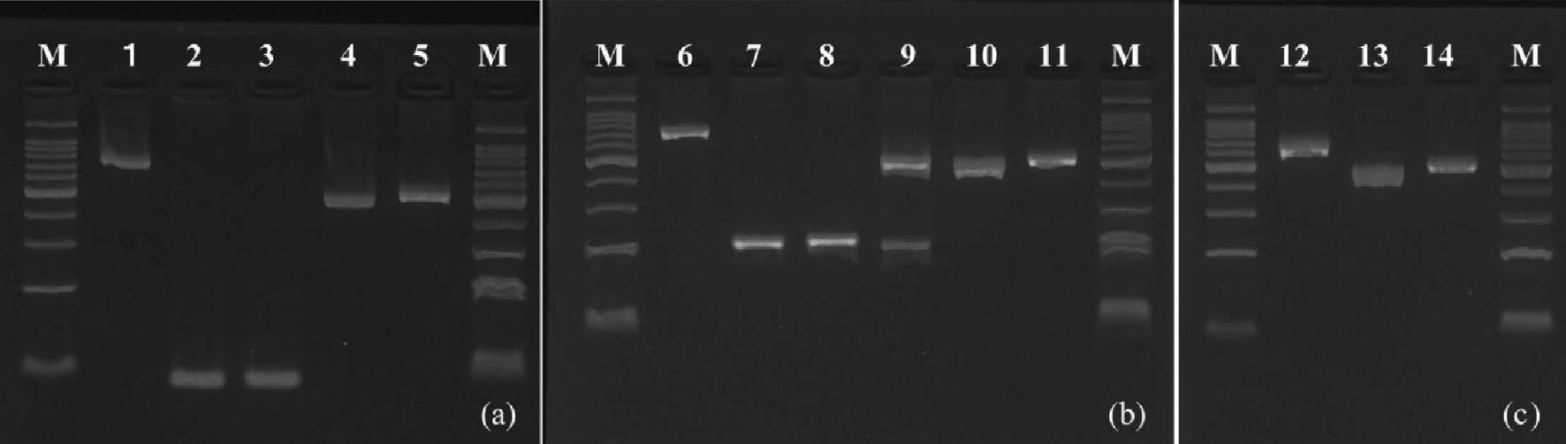
(**a**) Electrophoretic gel image of PCR products using common (1) and specific primers (2–5) for *Bombus cryptarum florilegus* and *B. terrestris*. The gel shows the DNA marker (M), DNA samples from the leg (1 and 2) and spermatheca (3 and 4) of *B. c.f.* queens, and DNA samples from the legs of *B. terrestris* queens (5) (spermatheca inseminated by conspecific male 3, and spermatheca inseminated by allospecific *B. terrestris* male 4). (**b**) Electrophoretic gel image of PCR products using common (6) and specific primers (7–11) for *B. hypocrita sapporensis* and *B. terrestris*. The gel shows the DNA marker (M), DNA samples from the leg (6 and 7) and spermatheca (7–10) of *B. h. s*. queens, and DNA samples from the legs of *B*. *terrestris* queens (11) (spermatheca inseminated by conspecific male 8, spermatheca inseminated by conspecific and allospecific *B. terrestris* males 9, and spermatheca inseminated by allospecific *B. terrestris* male 10). (**c**) Electrophoretic gel image of PCR products using common (12) and specific primers (13 and 14) for *B. terrestris*. The gel shows the DNA marker (M), as well as DNA samples from the leg (12 and 13) and spermatheca (14) of *B. terrestris* queens.

We observed overwrapped double peaks in the chromatograms of the mitochondrial DNA *COXI* gene sequences only in the DNA samples extracted from the spermathecae of *B. h. sapporensis* queens. Diagnostic PCR analyses of these samples revealed that 1.6% of the spermathecae in *B. h. sapporensis* queens contained DNA from both *B. h. sapporensis* and *B. terrestris* (Table 1 and Fig. 5).

## Discussion

Our GC-EAD analyses and behavioral tests provided evidence of cross-activities in sex pheromones between *B. terrestris* and two native Japanese bumblebee species, *B. h. sapporensis* and *B. c. florilegus*. The attractiveness of ethyl dodecanoate and 2,3-dihydrofarnesol to virgin *B. terrestris* queens, and only ethyl dodecanoate to *B. terrestris*, *B. h. sapporensis*, and *B. c. florilegus* queens, was demonstrated. Scent-marking with odorants produced by the cephalic part of the LG is a common behavior among male bumblebees to attract conspecific queens^17,18^. Each bumblebee species has its own species-specific blend of scent-marking components^19^. Although it is believed that scent-marker pheromones play a role in reproductive isolation, and that the common presence of ethyl dodecanoate and 2,3-dihydrofarnesol in the LG leads to cross-mating between native and non-native species^13^, to the best of our knowledge, the present study is the first to demonstrate their effects on queens’ behavior. Our findings indicate that escaped *B. terrestris* males mate with virgin queens of native species in Nemuro, Hokkaido, Japan, suggesting that the similarity in male sex pheromones between native and non-native bumblebees is a key factor in cross-mating the field. Although a common sex pheromone suggests the potential for cross-mating between *B. h. sapporensis* and *B. c. florilegus*, no cross-mating between native bumblebees was observed in this study. We speculate that due to differences in their reproductive seasons and strategies, the virgin queens and males of the two native bumblebee species rarely encounter each other in the field.

DNA analyses confirmed the occurrence of cross-mating between native and non-native species in the Nemuro Peninsula. This may be attributed to DNA sample contamination or the possibility of queens being inseminated by *B. h. sapporensis* and *B. terrestris* males. Although temperate *B. c. florilegus* queens are monandrous^12^, *B. h. sapporensis* and *B. terrestris* exhibit both monandrous and polyandrous behaviors in Japan^20^. It has been reported that 34.0% of *B. h. sapporensis* queens are polyandrous. Interestingly, native bumblebee males did not mate with *B. terrestris* queens, potentially due to the territorial dominance of *B. terrestris* males during premating behavior involving scent-marking^17,18^. This reproductive interference likely leads to a decline in native bumblebee populations because native queens that mate with *B. terrestris* do not produce viable offspring owing to the arrested embryonic development of laid eggs^10,11^.

The decline of native bumblebee populations in Japan, particularly those of *B. h. sapporensis* and *B. c. florilegus*, has been increasing due to the rapid naturalization of *B. terrestris*, resulting in habitat degradation and the fragmentation of *B. c. florilegus* populations^15^. Indeed, a recent study highlighted the fragmented population and reduced genetic diversity of *B. c. florilegus* in the Nemuro and Notsuke Peninsulas^14^. These findings, along with our results, underscore the need for immediate conservation measures to protect native bumblebees in the Nemuro Peninsula. Furthermore, future studies should consider control methods for feral *B. terrestris*.

## Methods

### Bumblebees

**Figure 6:**
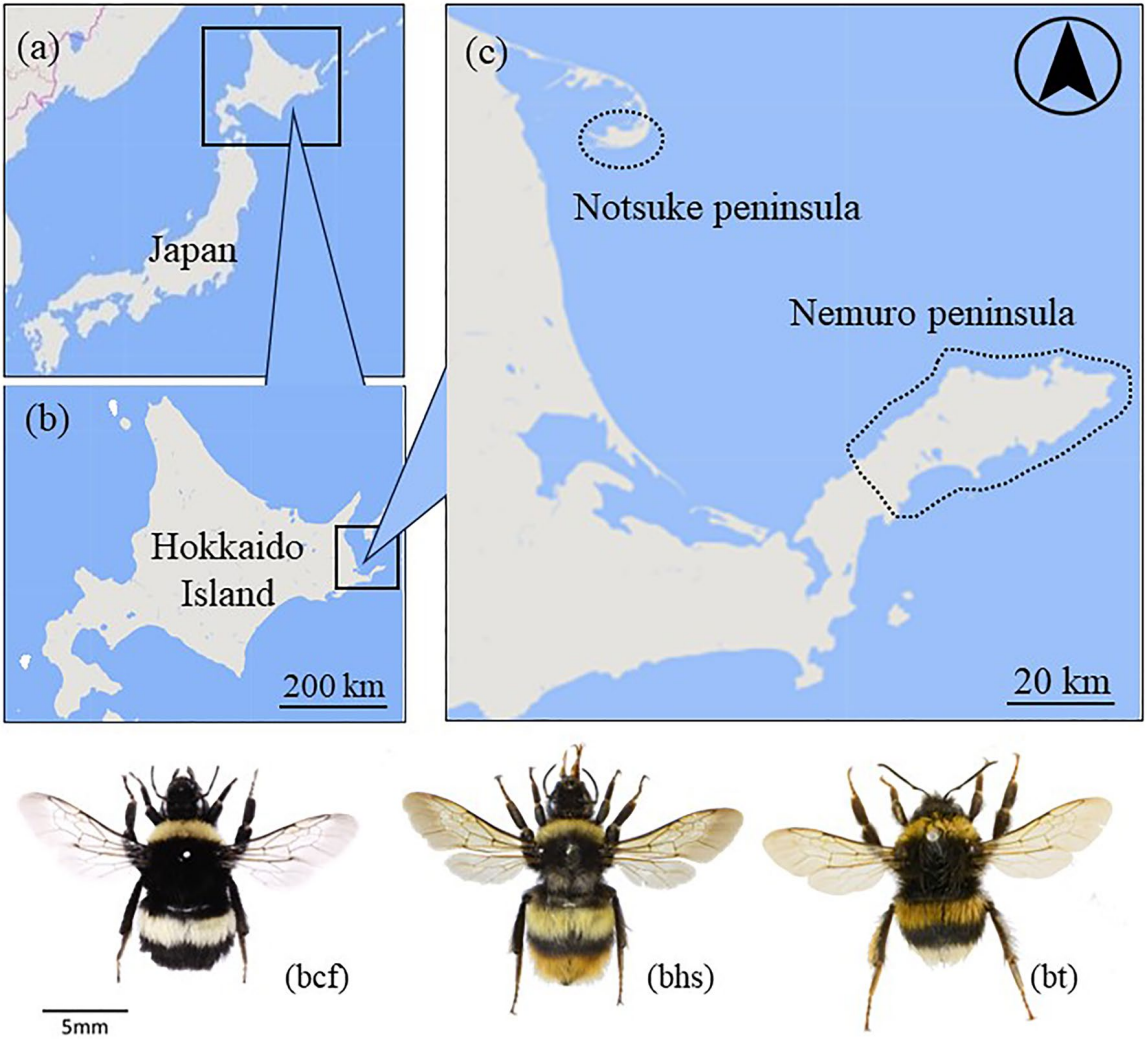
Distribution map of *Bombus* species sampling locations in Japan (**a**). *Bombus cryptarum florilegus* (bcf) is distributed only in the Nemuro and Notsuke Peninsulas (**c**), marked by dashed lines. *Bombus hypocrita sapporensis* (bhs) is distributed across Hokkaido Island (**b**), indicated by white and gray shading. *Bombus terrestris* (bt) is widely present and becoming naturalized across Hokkaido Island, indicated by gray shading. The dashed area represents the overlapping distributions of three *Bombus* species. Map created using, Vactor trial release (Geospatial Information Authority of Japan), https://maps.gsi.go.jp/vector/.

Virgin queens and males of *B. h. sapporensis* and *B. c. florilegus* were obtained from laboratory-reared colonies, unless otherwise specified. These colonies were established using mated queens collected from the Nemuro Peninsula, Hokkaido Island, Japan, between June 2011 and 2012 (Fig. 6). Additionally, a commercial colony of *B. terrestris* L. was purchased from Agrisect Inc. (Inashiki, Ibaraki, Japan). All colonies were reared in an air-conditioned room at 28 °C under constant darkness with a diet of pollen and a 55% sugar solution. Electrophysiological analyses and behavioral tests were conducted using 7-day-old males and 7–11-day-old new queens.

Between 2009 and 2019, 641 queens of *B. c. florilegus*, *B. h. sapporensis*, and *B. terrestris* were collected from the Nemuro Peninsula, Hokkaido Island, Japan (Fig. 6). The collection details are provided in Table 1. For DNA analysis, queens were preserved in 99% ethanol and stored at − 20 °C.

### Sample preparation for chemical analysis

Sample preparation for the analysis of male sex pheromones followed the method described in our previous study^13^. Male bumblebees were frozen at − 40 °C before chemical analysis and dissection to obtain LGs from the head. The LGs were placed in 4-mL vials, which were sealed with aluminum foil. Volatile components were extracted from the crushed LGs using solid-phase microextraction (SPME) headspace sampling for 10 min. The SPME device (SUPELCO; Sigma-Aldrich, St. Louis, Missouri, USA) included a fused-silica fiber coated with polydimethylsiloxane (100-µm thickness).

### GC-EAD analysis

The recording technique for antennal responses followed our previous study^21^. The GC-EAD system (TAIYO Co., Tsukuba, Japan) was used to investigate the antennal responses of *B. terrestris*, *B. h. sapporensis*, and *B. c. florilegus* virgin queens to extracts from the male LGs of conspecific and allospecific individuals. Volatiles were detected and separated using a DB-5MS column equipped with a GC-7890A system (Agilent Technologies, USA). The column temperature was initially set at 120 °C and then increased to 250 °C at a rate of 10 °C/min. Helium flow (6.5 mL/min) was used in a splitless injector port (250 °C), and a flame ionization detector port (300 °C) was used with a combination of hydrogen gas and air flow (30 mL/min and 400 mL/min, respectively).

Silver electrodes filled with conductive gel (Aquasonic; Parker Laboratories, USA) were connected to an amplifier. Antennae were extracted from live new queens using tweezers and positioned between the silver electrodes. Each analysis was repeated five times. Compounds that elicited responses in the antennae were considered EAD-active compounds.

To identify the EAD-active compounds, LG extracts were analyzed using gas chromatography–mass spectrometry following the same method used in GC-EAD analyses. Detected peaks were compared against the mass spectral database (Wiley 229), and presumed compounds were identified by comparing retention times and mass spectra with the respective chemical standards: 97% ethyl dodecanoate (Sigma-Aldrich) and 97% 2,3-dihydrofarnesol (TAIYO Co.). The identification 2,3-dihydrofarnesal was based on a comparison of the obtained mass spectra with those in the Wiley library and previously published data^16^.

### Behavioral experiment

Bioassays of queen bumblebees, used to assess their response to conspecific or allospecific male LG extracts, followed methods described previously^21,22^. In the behavioral experiment, a Y-tube olfactometer comprising a single long arm (length, 35.0 cm; diameter, 3.5 cm) and two short arms (length, 15.0 cm; diameter, 3.5 cm) was used. Glass cylinders (length, 10.0 cm; diameter, 2.5 cm) containing the test substances were connected to the end of the shorter arms using silicone tubing. The test substances included 2 μL of 0.001% ethyl dodecanoate (ED), 2,3-dihydrofarnesol (DF), a mixture of ethyl dodecanoate and 2,3-dihydrofarnesol (EFM) dissolved in pentane, as well as LG extracts of *B. terrestris* (*Bt*L), *B. h. sapporensis* (*Bhs*L), *B. c. florilegus* (*Bcf*L), and pentane alone. These substances were applied to a piece of filter paper in the glass cylinder. The LG extracts were obtained by immersing male LGs in 1 mL of pentane for 20 min. Both glass cylinders were connected to an air pump via silicone tubes of equal length, with air forced into each cylinder at a rate of 3 mL/min through a single inlet.

In total, 20, 16, and 10 virgin queens of *B. terrestris*, *B. h. sapporensis*, and *B. c. florilegus*, respectively, were included in the bioassays. Each queen was released into a plastic chamber (15 × 10 × 10 cm) connected to the long arm of the Y-tube. If a bee chose one of the shorter arms within 5 min, it was considered to have ‘‘chosen’’ the corresponding odorant. Bees that did not make a choice within this timeframe were excluded from the analysis. The experimental sequence consisted of choices between empty (E) vs. pentane, followed by *Bt*L vs. pentane, *Bhs*L vs. pentane, *Bcf*L vs. pentane, ED vs. pentane, DF vs. pentane, and EFM vs. pentane. To prevent potential bias toward a specific arm of the Y-tube, the positions of the treatment (*Bt*L, *Bhs*L, *Bcf*L, ED, DF, and EFM) and control (pentane) were changed after each run. The design of the Y-tube apparatus is shown in Supplementary Fig. 7.

### Statistical analysis

Data obtained from the Y-tube experiment were analyzed using the binomial test. *P* values for multiple comparisons were corrected using Holm’s method.

### DNA extraction from individuals and spermathecae

Genomic DNA from queens was extracted from the hind legs using the DNeasy Blood & Tissue Kit (QIAGEN). Sperm present in the spermathecae of queens was collected and processed as described previously^23^. Spermathecae were dissected in 1 × phosphate-buffered saline solution, and sperm clumps were separated from the membranes using insect pins. Genomic DNA from the male that mated with a queen was extracted from these sperm clumps using the DNeasy Blood & Tissue Kit (QIAGEN).

### Mitochondrial DNA sequence analysis

Fragments of the mitochondrial *COXI* gene, commonly used as a DNA barcoding region, were amplified via PCR using the primer pair COIF (HCO2198_t1: 5ʹ-TGTAAAACGACGGCCAGTGGTCAACAAATCATAAAGATATTGG-3ʹ) and COIR (LCO1490_t1: 5ʹ-CAGGAAACAGCTATGACTAAACTTCAGGGTGACCAAAAAATCA-3ʹ)^24^. The PCR amplifications consisted of an initial denaturation step at 98 °C for 2 min, followed by 30 cycles of denaturation at 98 °C for 10 s, annealing at 50 °C for 30 s, extension at 72 °C for 1 min, and a final extension at 72 °C for 10 min. *ExTaq* DNA polymerase (Takara, Otsu, Japan) was used for amplifications in a thermal cycler (Takara). PCR products were purified using ExoSAP-IT (USB Corporation, Cleveland, OH). Cycle sequencing reactions were performed using both primers and BigDye Terminator v.3.1 (Applied Biosystems). After product purification, sequencing was performed using an ABI 3130xl sequencer (Applied Biosystems). The obtained sequences were aligned using the GENETYX program (GENETYX, Tokyo, Japan), and a BLAST search was conducted for homology analysis.

### Determination by diagnostic PCR

Interspecific copulation between *B. c. florilegus*, *B. h. sapporensis*, and *B. terrestris* was confirmed through PCR and electrophoresis. Diagnostic PCR was conducted using bumblebee common and species-specific primers designed based on the *COI* gene sequences of *B. c. florilegus*, *B. h. sapporensis*, and *B. terrestris*, respectively^25^ (Table 2). The universal primer pair for DNA barcoding^24^ served as a positive control for DNA quality evaluation. The DNA used in the sequence analysis served as the template DNA. PCR was performed using the following cycle parameters: an initial denaturing step of 2 min at 95 °C, followed by 40 cycles of 15 s at 95 °C, 30 s at 50–58 °C, and 60 s at 72 °C. The final elongation step was extended to 10 min. HiDi DNA polymerase (myPOLS Biotec, Konstanz, Germany) was used in a thermal cycler (Takara). The PCR products were electrophoresed on 4% PrimeGel™ Agarose PCR-Sieve agarose gels (Takara) and visualized under ultraviolet light using Midorigreen Direct DNA Stain solution (NIPPON Genetics, Tokyo, Japan).

**Table 2:** Primer sequences and target *Bombus* species.

Primer name	Specificity	Sequence (5′–3′)	PCR product (bp)	MtDNA *COXI* gene position	Accession number
Bombus common forward	Bumblebee common	CAAATTATTATAAATGAAAGAGG	–	760	–
Bombus common reverse	Bumblebee common	TAATTATTTAATCATTCAAG	725	1484	–
Bcf specific reverse	*Bombus cryptarum*	CTCACACAATAAACCCTAAG	100	859	LC695022
Bhs specific reverse	*Bombus hypocrita*	GGTATGTAGCTAATCATCTGAAG	217	1288	LC695025
Bt specific reverse	*Bombus terrestris*	CTAAGAAATGTTGAGGGAAG	529	976	LC695021

### Nuclear DNA *ITS2* region sequence analysis

To determine the presence of interspecific mating queens, we also sequenced the nuclear DNA *ITS2* region. Randomly selected queens that were determined to have undergone interspecific mating based on diagnostic PCR for mitochondrial DNA, along with 20 queens per species determined to have undergone intraspecific mating, were analyzed. The *ITS2* region was sequenced using two primers and experimental conditions developed previously^26^. The *ITS2* sequence lengths of *B. h. sapporensis*, *B. c. florilegus*, and *B. terrestris* were approximately 2000 bp. The PCR fragments were ligated into a pUC19 vector and transformed into *Escherichia coli* JM109 competent cells (Takara, Otsu, Japan). In total, 360 recombinant colonies were picked and individually suspended in 20 μL of TE buffer. The inserts were amplified through PCR using M13-RV and M13–47 primers for the multicloning site of pUC19 (Takara, Otsu, Japan) following the manufacturer’s instructions. Three internal primers common to these bumblebees were designed for sequencing (BombusITS2_588: 5ʹ-GCAGGTTTTCGATGAGCACG-3ʹ; BombusITS2_1103: 5ʹ-ACGTTCGTCGGAAATCGTAC-3ʹ; BombusITS2_1474: 5ʹ-GTTGGTCATCCCATGCCTTT-3ʹ). The sequencing analysis method was the same as that used for sequencing the mitochondrial DNA *COXI* gene, as described above.

## Supplementary Information

Supplementary Information.

## Data Availability

Mitochondrial DNA sequences of three *Bombus* species are available in the DNA Data Bank of Japan: *Bombus cryptarum*: http://getentry.ddbj.nig.ac.jp/getentry/na/LC695021/?filetype=html; *Bombus hypocrita*: http://getentry.ddbj.nig.ac.jp/getentry/na/LC695025/?filetype=html; *Bombus terrestris*: http://getentry.ddbj.nig.ac.jp/getentry/na/LC695022/?filetype=html. Additionally, nuclear DNA *ITS2* sequences of three *Bombus* species are available in the DNA Data Bank of Japan: *Bombus cryptarum*: http://getentry.ddbj.nig.ac.jp/getentry/na/LC769002/?filetype=html; *Bombus hypocrita*: http://getentry.ddbj.nig.ac.jp/getentry/na/LC769003/?filetype=html; *Bombus terrestris*: http://getentry.ddbj.nig.ac.jp/getentry/na/LC769004/?filetype=html.
