# Supplementary material for: Cross-mating between the alien bumblebee Bombus terrestris and two native Japanese bumblebees, B. hypocrita sapporensis and B. cryptarum florilegus, in the Nemuro Peninsula, Japan
Source: Sci Rep. 2023 Jul 17;13:11506. doi: 10.1038/s41598-023-38631-7 (PMC10352366; doi:10.1038/s41598-023-38631-7)
Supplement: Supplementary file 1 — Supplementary Information. [file 41598_2023_38631_MOESM1_ESM.docx]

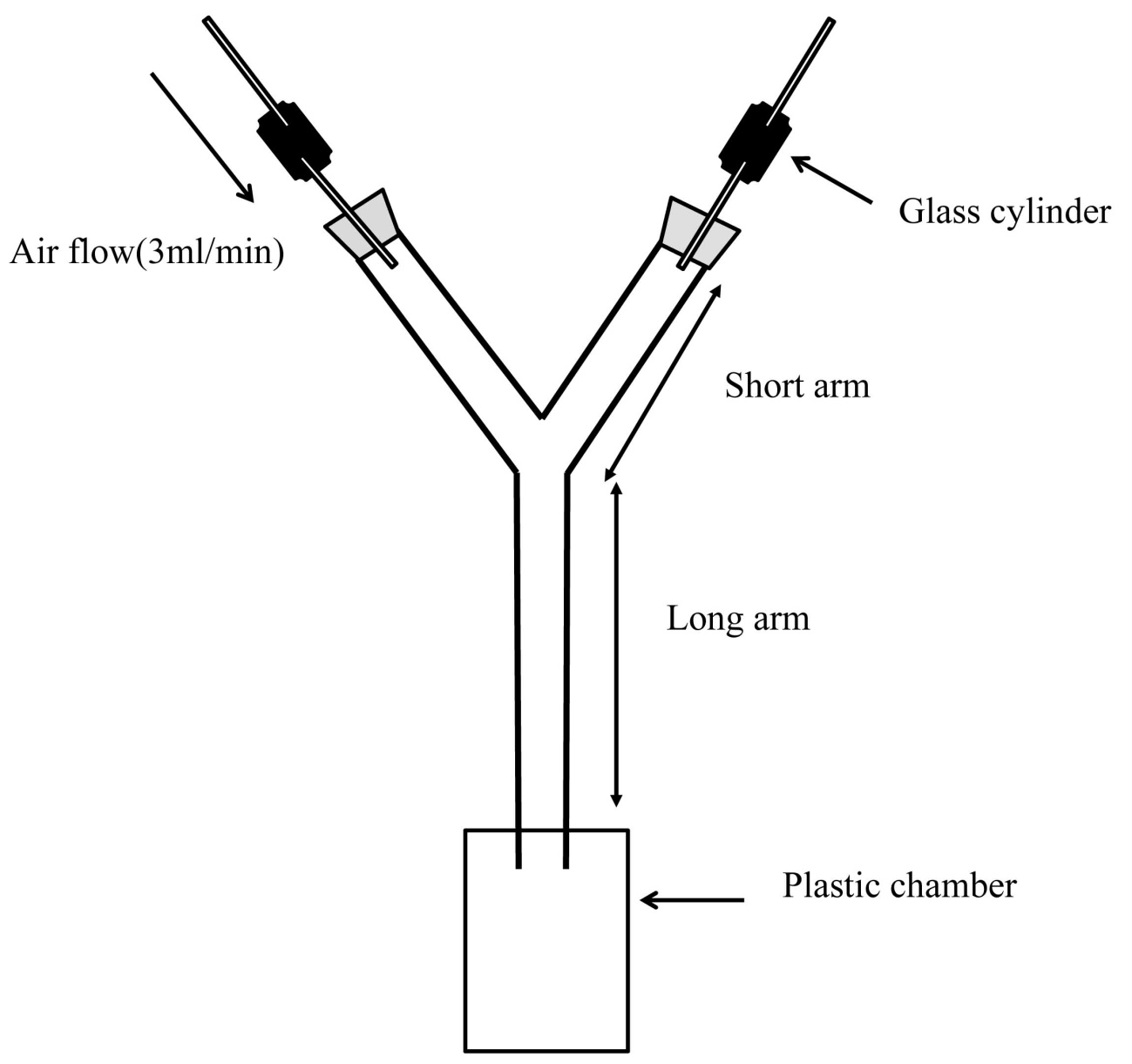


Figure 7

The design of the Y-tube apparatus used in the pheromone attraction experiments.

Table 3 Frequencies of intra- and interspecific mating in queens of *Bombus cryptarum florilegus*.

| Year | *n* | Number of the mate male in spermathecae | | | | Unmated* |
| --- | --- | --- | --- | --- | --- | --- |
|  |  | *Bcf* | *Bhs* | *Bt* | *Bhs* and *Bt* |  |
| 2009 | 15 | 11 | 0 | 2 | 0 | 2 |
| 2010 | 12 | 8 | 0 | 2 | 0 | 2 |
| 2011 | 13 | 11 | 0 | 0 | 0 | 2 |
| 2012 | 14 | 11 | 0 | 3 | 0 | 0 |
| 2013 | 15 | 12 | 0 | 1 | 0 | 2 |
| 2014 | 11 | 10 | 0 | 0 | 0 | 1 |
| 2015 | 10 | 8 | 0 | 0 | 0 | 2 |
| 2016 | 14 | 11 | 0 | 0 | 0 | 3 |
| 2017 | 13 | 8 | 0 | 3 | 0 | 2 |
| 2018 | 11 | 9 | 0 | 1 | 0 | 1 |
| 2019 | 13 | 10 | 0 | 1 | 0 | 2 |
| Total | 141 | 109 | 0 | 13 | 0 | 19 |
| *The unmated queen indicates the absence of sperm in the spermatheca. | | | | | | |

Table 4 Frequencies of intra- and interspecific mating in queens of *Bombus hypocrita sapporensis*.

| Year | *n* | Number of the mate male in spermathecae | | | | Unmated* |
| --- | --- | --- | --- | --- | --- | --- |
|  |  | *Bhs* | *Bcf* | *Bt* | *Bhs* and *Bt* |  |
| 2015 | 50 | 44 | 0 | 4 | 0 | 2 |
| 2016 | 50 | 48 | 0 | 2 | 0 | 0 |
| 2017 | 50 | 43 | 0 | 4 | 1 | 2 |
| 2018 | 50 | 45 | 0 | 3 | 1 | 1 |
| 2019 | 50 | 44 | 0 | 4 | 0 | 2 |
| Total | 250 | 224 | 0 | 17 | 2 | 7 |
| *The unmated queen indicates the absence of sperm in the spermatheca. | | | | | | |

Table 5 Frequencies of intra- and interspecific mating in queens of *Bombus terrestris*.

| Year | *n* | Number of the mate male in spermathecae | | | Unmated* |
| --- | --- | --- | --- | --- | --- |
|  |  | *Bt* | *Bcf* | *Bhs* |  |
| 2015 | 50 | 49 | 0 | 0 | 1 |
| 2016 | 50 | 48 | 0 | 0 | 2 |
| 2017 | 50 | 49 | 0 | 0 | 1 |
| 2018 | 50 | 48 | 0 | 0 | 2 |
| 2019 | 50 | 49 | 0 | 0 | 1 |
| Total | 250 | 243 | 0 | 0 | 7 |
| *The unmated queen indicates the absence of sperm in the spermatheca. | | | | | |
